# The Influence of Dental Status and Blood Parameters Characterizing Endogenous Intoxication on the Timing of Childbirth

**DOI:** 10.3390/medicina60071176

**Published:** 2024-07-19

**Authors:** Maria Hakobyan, Gayane Manrikyan, Marina Markaryan, Izabella Vardanyan, Mikayel Manrikyan

**Affiliations:** 1 Department of Prosthodontics, Yerevan State Medical University (YSMU), Koryun Str. 2, Yerevan 0002, Armenia; hakobyan_maria@yahoo.com; 2 Department of Therapeutic Dentistry, Yerevan State Medical University (YSMU), Koryun Str. 2, Yerevan 0002, Armenia; gana-stom@yandex.ru; 3 Department of Pediatric Dentistry and Orthodontics, Yerevan State Medical University (YSMU), Koryun Str. 2, Yerevan 0002, Armenia; iza.vard67@gmail.com (I.V.); dr.manrikyan@mail.ru (M.M.)

**Keywords:** chronic oral focus of infection, periodontal diseases, preterm birth, blood cell parameters

## Abstract

*Background and Objectives*: Epidemiological and microbiological–immunological studies have led to the conclusion that periodontal disease may be a risk factor for preterm birth. The aim of this study was to investigate and identify the relationship of some hematological cellular biomarkers characterizing the chronic oral focus of infection with pregnancy outcomes and their impact on those outcomes. *Materials and Methods*: Clinical and laboratory tests were conducted on 100 pregnant women, grouped by full-term or preterm births, with the assessment of the following markers: DMF, CPI and PIRI, PHP, microbiological examination of periodontal pockets and amniotic fluid, WBS count, WBCSI, LGI, and NMR. A statistical analysis was carried out with SPSS Statistics version 19.0. *Results*: Women with preterm labor had higher-grade caries (CSL > 0.3), while women with full-term childbirth had moderate caries (CSL < 0.3). A satisfactory level of oral hygiene efficiency was found in 50% (group 1) and 38.1% (group 2) of the expectant mothers. The periodontal status by the PIRI showed tissue lesions in 20.7% (group 1) and 92.9% (group 2) of the women. The WBCSI was 2.27 ± 0.82 and 2.15 ± 0.68, the NMR was 9.29 ± 5.119 and 11.62 ± 7.78, and the LGI was 3.54 ± 1.1 and 3.73 ± 0.81 in groups 1 and 2, respectively. Comparative analysis of bacterial contamination of the amniotic fluid revealed the predominance of *Fusobacterium nucleatum* (64.3%), *Tannerella forsythia* (57.1%), *Prevotella intermedia* (50%), *Porphyromonas gingivalis* (57.1%), *Staphylococcus aureus* (45.2%), and *Candida albicans* (50%) in women with premature birth. *Conclusions*: In women with preterm birth, the values of the indices characterizing a chronic oral focus of infection evoke more significant correlations with the timing of delivery, which indicates the significant role of an oral focus of infection. The presence of microbial invasion of amniotic fluid may indicate the role of periodontopathogenic bacteria in pregnant women diagnosed with a risk of preterm birth.

## 1. Introduction

Inflammatory diseases of the periodontal tissue are one of the most pressing problems in modern dentistry. The results of many clinical, experimental, and epidemiological studies indicate that infectious diseases of the oral cavity tissue (especially inflammatory lesions of the periodontium) form the so-called “chronic odontogenic foci of infection”, which are risk factors for the development of numerous systemic diseases or for the worsening of their clinical course [1,2,3]. Chronic inflammation in the oral cavity, including asymptomatic (silent) inflammation, is also a source of chronic intoxication in the body of a pregnant woman [4,5]. Systemic edema, manifested by increased vascular blood flow along with an increase in the gingival fluid flow rate, at least facilitates the gingival alterations that occur during pregnancy [6]. Women complain of mouth discomfort and bleeding gums, which can lead to pregnancy gingivitis [7,8].

The pathogenic microorganisms, along with their metabolic byproducts, enter the bloodstream and lymphatic system and penetrate all organs and systems of the body of the expectant mother, as well as the amniotic fluid surrounding the fetus. All of these may lead to a loss of pregnancy and spontaneous miscarriage or provoke a premature birth and the birth of a child with a low body weight. The health status of a pregnant woman affects the antenatal processes of mineralization of the enamel of deciduous teeth, and the sanitation of the oral cavity of the pregnant woman is antenatal prevention of dental caries in the future baby [9,10,11,12].

Periodontitis is a chronic inflammatory illness with a multifactorial etiology that is characterized by the progressive destruction of the supporting structures of teeth [13,14,15]. According to Beck and Offenbacher, the traditional classification of mild, moderate, and severe periodontitis or other methods of measuring the severity of the disease, such as loss of bone attachment, are not the best markers for determining the risk of systemic inflammation secondary to periodontal disease. Bleeding on probing may be the most important clinical indicator of an ongoing active inflammatory process and may best reflect subgingival ulceration and represent a potential route for systemic access to the adjacent subgingival flora. Probing depth can provide information about how much of the subgingival area is exposed to microbial burden. The Periodontal Infection Risk Index (PIRI) is one of the most notable new concepts. This index has a biological basis similar to that proposed by Beck and Offenbacher, i.e., it is related to the amount of ulcerated subgingival area exposed to bacterial load and poses a potential systemic threat in patients with periodontal disease [16,17]. Epidemiological and microbiological–immunological studies have led to the conclusion that periodontal disease may be a distinct risk factor for cardiovascular, cerebrovascular, and respiratory disorders, as well as for preterm birth of low birth weight (LBW) infants [18,19,20]. The 29th World Health Assembly (1976) adopted an international definition of LBW for infants with a birth weight of “less than 2500 g” (up to and including 2499 g) [21]. According to the World Health Organization (WHO), preterm birth is considered if a live birth occurs at less than 37 weeks of gestation [22].

In a healthy organism, the counts of individual forms of white blood cells are in a constant percentage ratio. The cause of leukocytosis in inflammatory diseases is the stimulation of the leukopoietic function of hematopoietic organs as a result of the action of specific pathogens and inflammatory factors [23]. Assessment of the proportion of different types of white blood cells (such as the lymphocyte-to-granulocyte index, white blood cell shift index, neutrophil-to-lymphocyte ratio, etc.) based on the results of the complete blood count plays a crucial role in the diagnosis and monitoring of endogenous intoxication caused by chronic infection foci. Neutrophils, Neutrophils, one of the most abundant type of white blood cell, are the classic cells of the inflammatory pathway that link the innate and adaptive parts of the immune system and are often considered a double-edged sword in the immune response [24]. Many recent studies have scrutinized the role of total and differential serum white blood cell counts in various chronic conditions, including periodontitis [24]. The white blood cell ratios, namely the neutrophil-to-lymphocyte ratio (NLR) and platelet–lymphocyte ratio (PLR), have already proven useful in the diagnosis and prognosis of several chronic inflammatory diseases such as diabetes mellitus, cardiovascular diseases, chronic lung diseases, and several types of cancer [25,26]. Similarly, the use of leukocyte-to-monocyte ratio (LMR) is being studied as a diagnostic factor. The importance of LMR in determining prognosis in cardiovascular diseases and solid tumors is well established [24,27,28]. These markers have recently been proposed as simple, rapid, cost-effective, and readily available biomarkers of systemic inflammation and have proven to be particularly useful in low- and middle-income countries.

However, little is known about the association of periodontitis with NLR, PLR, and LMR, which may not only serve as potential biomarkers of systemic inflammation but also help in the diagnosis/screening of severe periodontitis and predicting its prognosis.

Ensuring proper oral hygiene is crucial in mitigating the development and progression of common oral conditions, such as dental caries and periodontal disease. One of the primary strategies for preserving optimal oral hygiene is self-administered mechanical plaque control, specifically through regular tooth brushing routines [29].

The scientific literature periodically addresses the topic of the impact of common inflammatory periodontal lesions and markers of an oral chronic infectious focus on the course and outcome of pregnancy; however, there is a lack of studies that attempt to identify, explain, and justify the etiological factors, the mechanisms of influence of this phenomenon, and the principles of its diagnosis through clinical and laboratory tests.

The aim of this study was to investigate and identify the relationship between hematological cellular biomarkers that characterize the chronic oral focus of infection and pregnancy outcomes and their impact on those outcomes.

## 2. Materials and Methods

During the period from 2020 to 2024, clinical and laboratory tests were carried out at the Research Center of Maternal and Child Health Protection (Yerevan, Armenia) on 100 pregnant women in the third trimester of pregnancy (prenatal period), aged 20 to 40 years (average 28.02 ± 5.03). They were divided into 2 groups, depending on the term of delivery: full-term birth (n = 58, group 1) and preterm birth (n = 42, group 2). The Ethical Committee of Yerevan State Medical University (N 4/12.19) approved the study. In accordance with the Helsinki Declaration of 2008, written informed consent for participation in the study was obtained from all expectant mothers included in the study.

A general stomatologist performed a periodontal examination using a disposable mirror and periodontal probe under sufficient natural light lighting.

Caries intensity was determined using the Decayed, Missing, and Filled (DMF) Index, and caries severity level was assessed according to the World Health Organization (WHO) criteria [30].

The condition of periodontal tissues was assessed by the Community Periodontal Index (CPI) across six sextants (17/16, 11, 26/27, 36/37, 31, and 46/47) using a periodontal probe. The extent and severity of periodontal tissue lesions were determined [30].

The risk of periodontal-systemic complications was assessed by the PIRI. The PIRI allows evaluation of the pumping effect—the pathway of penetration of biofilm components under the gum from the thin and predominantly bleeding epithelium of periodontal pockets into the blood. This phenomenon cannot be detected using classical periodontal indicators, which can only estimate the level of tissue destruction for epidemiological studies. The examination included determining the depth of probing and damage to the furcations of all teeth. Probing was carried out on all teeth in 4 quadrants.

The total score shows the rating PIRI of the patient (Table 1).

Depending on the total PIRI score, patients were categorized into three groups: patients with a PIRI equal to 0 (low risk of periodontal–systemic complications), patients with a PIRI ≤ 1 to ≤5 (intermediate risk of periodontal–systemic complications), and patients with a PIRI ≤ 6 to ≤10 (high risk of periodontal–systemic complications) [16].

The Patient Hygiene Performance (PHP) Index, according to Podshadley and Haley (1968), was used to evaluate oral hygiene efficiency, with scores recorded for six index tooth surfaces: the buccal/lingual surfaces of both first molars of the upper jaw, the right upper and left central incisors of the lower jaw, as well as the lingual surfaces of first molars of both lower jaws [30,31].

Parameters characterizing endogenous intoxication in pregnant women were investigated.

Laboratory analysis. A microbiological examination established the composition of the microflora of gingival sulci, periodontal pockets, and amniotic fluid, as well as the differentiation of the microflora, which is important for diagnosis and the subsequent choice of medication treatment.

A collection of amniotic fluid was performed in women with preterm labor and normal birth with intact membranes or during a cesarean section.

Microbiological cultures under aerobic, micro-aerophilic, and anaerobic conditions were carried out using the following main nutrient media (all from Liofilchem, Roseto degli Abruzzi, Teramo, Italy): Columbia agar, Chocolate agar, Schaedler agar, Sabouraud CAF agar, Endo agar, Mannitol salt agar, and MRS agar. Cultivation of aerobic bacteria was carried out for 1 to 3 days at 37 °C, while fungi were cultivated at 30 °C. The cultures of microaerophiles and anaerobe bacteria were carried out on an aerostat produced by bioMerieux (bioMerieux, Craponne, France).

Determination of some hematological cellular parameters characterizing endogenous intoxication was carried out using the following indices: white blood cell (WBC) count and left shift of white blood cell shift index (WBCSI), lymphocyte-to-granulocyte index (LGI), and neutrophil-to-monocyte ratio (NMR).

The lymphocyte-to-granulocyte index (LGI) was calculated using the following formula:

LGI = LYM × 10/(Ml + metaMl + bN + sN + E + B),

where LYM—lymphocytes; Ml—myelocytes; metaMl—metamyelocytes; bN—banded neutrophils; sN—segmented neutrophils; E—eosinophils; B—basophils in the leukocyte formula of peripheral blood. The normal mean ± standard deviation value of LGI is 4.56 ± 0.37. A tendency toward an increase in LGI indicates the presence of autointoxication in the body.

The white blood cell shift index (WBCSI) is a marker of the body’s reactivity during acute inflammation and an indicator of the activity of the inflammatory process and impaired immunologic reactivity. The WBCSI is calculated by the ratio of the total counts of eosinophils, basophils, myelocytes, metamyelocytes, and banded and segmented leukocytes to the sum of monocytes and lymphocytes. Normally, WBCSI is equal to 1.96.

The neutrophil-to-monocyte ratio (NMR) is calculated by the ratio of the total counts of metamyelocytes, myelocytes, and banded and segmented leukocytes to the number of monocytes. Normally, NMR is equal to 11.83.

Exclusion criteria included systemic comorbidities, such as diabetes mellitus, cardiovascular diseases, hypertension, severe anemia, genitourinary tract infections, as well as multiple pregnancies, preeclampsia or eclampsia, and gestational diabetes.

Statistical analysis was carried out using the IBM SPSS Statistics version 19.0 computer software. Mean values and standard deviations (SDs) of variables were calculated. Correlations between variables were calculated using Pearson’s correlation test. Differences between groups were tested using the paired samples *t*-test for normally distributed data. For each test, the *p*-value was set at the level of significance of 0.05.

## 3. Results

The mean ± SD gestational age in the full-term birth (group 1) and preterm birth (group 2) groups was 38.93 ± 1.1 and 32.8 ± 2.98 weeks, respectively, with a statistically significant intergroup difference (*t* = 12.67, *p* < 0.001). Most of the newborns in group 2 had significantly lower birth weight: 2076.2 ± 536.9 g versus 3293.9 ± 459.8 g in group 1 (*t* = 11.9, *p* < 0.001), indicating a significant increase in the incidence of LBW in preterm birth compared to full-term birth.

A satisfactory level of oral hygiene efficiency was found in 29 (50%) and 16 (38.1%) expectant mothers in groups 1 and 2, respectively. An unsatisfactory level of oral hygiene was observed in 9 (15.5%) and 23 (54.8%) pregnant women in groups 1 and 2, respectively. The study of oral hygiene habits showed that only 20 (34.5%) women in group 1 and 16 (38.1%) women in group 2 were regularly brushing their teeth twice a day.

The level of oral hygiene efficiency in both groups was within the satisfactory range: 0.98 ± 0.52 and 1.52 ± 0.58 in groups 1 and 2, respectively (the difference was statistically significant, *t* = −4.8, *p* < 0.001).

Most women (87%) in both groups had visited a dentist at least once, and 11% were on an ongoing stomatological treatment. The main problems for which they visited the stomatologist on their last visit were consultation (20%), endodontic treatment (17%), and extraction of decayed teeth (15%). The main reasons they did not see a stomatologist were: “I have no toothache” (38.5%), “I am pregnant” (23.1%), and “Financial reasons” (15.4%). A total of 23% of women did not want to answer the question.

The distribution of values of variables characterizing the lesion of the dentomandibular complex by groups is presented in Table 2.

The data provided in the table demonstrate that in group 2, the indicators of dental lesions significantly exceeded the same indicators obtained in group 1; the difference was statistically significant in all comparisons, except for the “Filling” and “Missing” components of the DMF index.

The overall prevalence of periodontal disease among pregnant women who visited the health center was 98.3%. Only one (1.7%) subject in group 1 had a CPI score of 0 (healthy periodontium), whereas none of the subjects in group 2 had a CPI score of 0.

Gingivitis was detected in 49 (49%) of the pregnant women examined.

A CPI score of 4 was detected in 1 (1.7%) woman in group 1 and in 10 (23.8%) women in group 2. Excluded sextants were detected in 15 (25.9%) and 16 (38.1%) subjects in groups 1 and 2, respectively. In group 2, the CPI values characterizing the clinical state of the periodontium were also distinguished by the most clinically pronounced criteria (Table 3).

The periodontal complex status, according to the PIRI, revealed periodontal tissue lesions in 20.7% (n = 12) and 92.9% (n = 39) of women in groups 1 and 2, respectively.

The total WBC count in both groups was within the physiologically normal range: 8.5 ± 2.18 and 9.5 ± 2.7 in groups 1 and 2, respectively (however, significantly higher in women of the preterm birth group: *p* = 0.046), and practically did not differ from average statistical values. The band neutrophil count in groups 1 and 2 was 8.3 ± 3.8 and 9.38 ± 3.9, respectively (*t* = −1.4; *p* = 1.8). Among the indices characterizing the presence of chronic and purulent inflammation in the body, the WBCSI was 2.27 ± 0.82 and 2.15 ± 0.68, the NMR was 9.29 ± 5.119 and 11.62 ± 7.78, and the LGI was 3.54 ± 1.1 and 3.73 ± 0.81, in groups 1 and 2, respectively.

In both study groups, the correlation analysis of the data yielded statistically significant direct (positive) or inverse (negative) correlations between the gestational age at delivery and the PHP index (r = −0.3, *p* < 0.001), the PIRI (r = −0.4, *p* < 0.001), and the birth weight (r = 0.8, *p* < 0.001).

The prevalence of ascending bacterial infection was 84.5% (n = 49) and 90.5% (n = 38) in groups 1 and 2, respectively.

In a comparative analysis of the group of women who gave birth prematurely, *Tannerella forsythia* was detected as the most common bacterium in the periodontal pockets (100% of cases) and in the amniotic fluid in 57.1% of cases (n = 24) (*p* < 0.001). *Porphyromonas gingivalis* (92.9%), *Prevotella intermedia* (85.7%), *Candida albicans* (81%), *Fusobacterium nucleatum* (78.6%), *Aggregatibacter* spp. (78.6%), and *Staphylococcus aureus* (54.8%) were also detected in the gingival fluid of women in group 2. The abovementioned bacteria were also found in 45.2–64.3% of cases in samples of amniotic fluid from patients who gave birth prematurely (group 2). A direct correlation was identified between gingival contamination and the presence of microorganisms in the amniotic fluid in women in this group (Table 4).

The percentage distribution and correlation of microorganisms in the gingival and amniotic fluids of pregnant women who gave birth at term (group 1) are summarized in Table 5.

## 4. Discussion

In group 1, no correlation was found between the PHP index, age, and delivery time, whereas in group 2, a significant positive correlation was found between the PHP index and age (r = 0.591, *p* < 0.001) and delivery time (r = 0.417, *p* = 0.006). In both groups, a strong correlation was found between the oral hygiene efficiency index and the DMF and CSL indices (*p* < 0.001). In both groups, the DMF index corresponded to moderate caries severity (according to WHO); the assessment of CSL showed that women with preterm labor had higher-grade caries (CSL > 0.3), while women with full-term childbirth had moderate caries (CSL < 0.3).

According to the average PIRI scores, the women in group 1 had a low risk of periodontal systemic complications (0.45 ± 0.9), whereas the women in group 2 were at medium risk (2.17 ± 0.85) (*t* =−9.7, *p* < 0.001).

When studying the qualitative composition of peripheral leukocytes, an increase in the band neutrophil counts was found in both groups, which is typical in the presence of foci of bacterial infection. The analysis of the WBC count in both groups showed the absence of significant (*p* > 0.05) differences in the counts of segmented neutrophils, lymphocytes, monocytes, and eosinophils. Some authors believe that defining one single indicator is not sufficient to reflect the health status of the patient; therefore, a comprehensive approach to the use of several indicators describing the same phenomenon is necessary [24]. In both groups, the values of hematological parameters characterizing endogenous intoxication were outside the normal range in almost all cases. Indicators characterizing the presence of chronic and purulent inflammation in the body (WBCSI) in both groups exceeded normal values and corresponded to a low-grade endogenous intoxication, but intergroup differences were not statistically significant (*t* < 2, *p* > 0.1). In the study groups, the NMR value was below the clinical norm, but no significant intergroup differences were found (*t* < 2, *p* = 0.09). The determination of the ratio of lymphocytes to granulocytes revealed that the blood LGI in all examined subjects was below the physiological norm (1.3 in group 1 and 1.2 in group 2). A decrease in this index, according to the literature, indicates infectious intoxication and an active, timely response of the immune system to an inflammatory process, which can be regarded as a favorable sign [32].

The analysis of correlations between individual indicators characterizing the clinical state of teeth, periodontium, and endogenous intoxication also indicates a clearer expression of these associations in the group with preterm labor. In particular, in this group of women (group 2), we revealed a medium or high correlation between the PHP index and both the age of laboring women and the gestational age at delivery. The childbirth term significantly correlated with the dental tartar index of the CPI, with the PIRI components of periodontal pocket lesions and furcations, and with the CSL score. Moderate and strong correlations were found between the CPI components “Pocket 4–5 mm” and “Pocket 6 mm and more” and the PIRI components “Periodontal pocket lesions” and “Furcations” in group 2. In the group of women with a normal term of labor (group 1), a correlation was observed between the CPI components “Pocket 4–5 mm” and “Pocket 6 mm and more” and the PIRI components “Periodontal pocket lesions” and “Furcations”.

While studying the oral microflora, the presence of pathogenic composition was found in all the groups observed. Pronounced growth of microorganism colonies was observed in all the groups. Both Gram-positive (*Peptostreptococcus*, *Peptococcus*, *Lactobacillus* spp., *Corynebacteria*, *α*- and *β*-hemolytic streptococci, and staphylococci) and Gram-negative (*Neisseria* spp., *Fuzobacterium* spp., and *Prevotella intermedia*) microorganisms were detected in the gingival crevicular fluid in all observation groups. The comparative analysis of bacterial contamination of the amniotic fluid of pregnant women revealed the following statistically significant pattern: in women with premature birth (group 2), the presence of *Fusobacterium nucleatum* (64.3%), *Tannerella forsythia* (57.1%), *Prevotella intermedia* (50%), *Porphyromonas gingivalis* (57.1%), *Staphylococcus aureus* (45.2%), and *Candida albicans* (50%) was predominant. It has been established that periodontal tissue complex diseases are associated with ascending bacterial infection, affect the timing of childbirth, and are placental markers of bacterial ascending infection [33,34,35,36,37].

## 5. Conclusions

Thus, in expectant mothers with full-term and preterm deliveries, an analysis of the values of hematological parameters characterizing endogenous intoxication shows that there are phenomena of infectious intoxication in the third trimester, especially noticeable in pregnant women with premature births. When comparing the same indicators in both groups, they are not separated by statistical significance. In women with preterm birth (as opposed to full-term birth), these indicators, as well as the values of the indices characterizing a chronic oral focus of infection, evoke more significant correlations with the timing of delivery, which additionally indicates the significant role of a chronic oral focus of infection. Due to the small number of participants in both groups, no significant association was found between maternal age and LBW. The early referral of pregnant women to a dentist for oral health education and guidance throughout pregnancy is warranted. The presence of microbial invasion of the amniotic fluid may indicate the role of periodontopathogenic bacteria in pregnant women diagnosed with a risk of preterm birth.

## Figures and Tables

**Table 1:** Definition of the PIRI.

Pocket Lesion	Score 1	Furcation Involvement	Score 2
<5 pockets 5–6 mm	1	<3 furcations of class I	1
≥5 pockets 5–6 mm	2	≥3 or <3 furcations of class I or II	2
<5 pockets 7–8 mm	3	>3 or <3 furcations of class II or III	3
≥5 pockets 7–8 mm	4	≥3 furcations of class III	4
<3 pockets ≥9 mm	5	-	-
≥3 pockets ≥9 mm	6	-	-
PIRI scores		score 1 + score 2	

**Table 2:** Indicators of hard dental tissue lesion in pregnant women with full-term or preterm delivery.

Indicators	Preterm Birth	Full-Term Birth	*t*	*p*-Value
Decay	5.43 ± 2.4	3.76 ± 1.82	−3.78	0.001
Filling	1.52 ± 1.25	1.55 ± 0.995	0.046	0.96 *
Missing	1.86 ± 1.14	1.67 ± 1.22	−0.78	0.44 *
DMF	8.81 ± 3.88	6.95 ± 2.7	−2.67	0.009
Caries severity level	0.31 ± 0.11	0.25 ± 0.97	−2.7	0.009

* *p* > 0.05, a statistically non-significant difference.

**Table 3:** Periodontal tissue status according to the CPI (by sextants) in pregnant women with full-term or preterm delivery.

Indicators	Preterm Birth	Full-Term Birth	*t*	*p*-Value
CPI 0	0.31 ± 0.78	1.66 ± 1.69	5.3	0.001
CPI 1	0.88 ± 0.99	1.62 ± 1.4	3.1	0.003
CPI 2	2.26 ± 0.86	2.05 ± 1.4	−0.9	0.36 *
CPI 3	1.88 ± 1.06	0.34 ± 0.8	−7.8	0.001
CPI 4	0.31 ± 0.6	0.03 ± 0.26	−2.76	0.008
CPI excluded	0.38 ± 0.5	0.28 ± 0.49	−1.06	0.29 *

* *p* > 0.05, a statistically non-significant difference.

**Table 4:** Distribution (%) and correlation of microorganisms in the gingival and amniotic fluids of pregnant women who gave birth prematurely (group 2).

	Amniotic Fluid	*Aggregatobacter* spp.	*Tannerella* *forsythia*	*Fusobacterium nucleatum*	*Prevotella intermedia*	*Porphyromonas gingivalis*	*Staph. aureus*	*Candida albicans*
Gingival Fluid								
*Aggregatobacter* spp.		0.498 (*p* = 0.001)	0.369 (*p* = 0.016)	-	-	-	-	-
*Fusobacterium nucleatum*		0.701 (*p* < 0.001)	-	-	-	-	-	-
*Prevotella intermedia*		-	-	0.406 (*p* = 0.008)	0.408 (*p* = 0.007)	0.334 (*p* = 0.031)	0.371 (*p* = 0.016)	-
*Porphyromonas gingivalis*		-	-	0.372 (*p* = 0.015)	-	0.32 (*p* = 0.039)	-	-
*Staph. aureus*		-	-	-	-	-	0.826 (*p* = 0.001)	-
*Candida albicans*		-	-	-	-	-	0.319 (*p* = 0.039)	0.485 (*p* = 0.001)

**Table 5:** Distribution (%) and correlation of microorganisms in the gingival and amniotic fluids of pregnant women who gave birth at term (group 1).

Microorganisms	Gingival Fluid	Amniotic Fluid	*p*-Value	r
*Aggregatobacter* spp.	69%	39.7%	<0.001	0.544 *
*T. forsythia*	63.8%	32.8%	<0.001	0.526 *
*Fusobacterium nucleatum*	60.3%	34.5	<0.001	0.588 *
*P. intermedia*	55.2%	24.1	<0.001	0.508 *
*Porphyromonas gingivalis*	53.4%	32.8	<0.001	0.651 *
*Staph. aureus*	51.7%	24.1	<0.001	0.545 *
*Candida albicans*	43.1%	29.3	0.01	0.663 *

* *p* < 0.05.

## Data Availability

The datasets presented in this article are not readily available because the data are part of an ongoing study.

## References

[1] Alnasser B.H., Alkhaldi N.K., Alghamdi W.K., Alghamdi F.T. (2023). The Potential Association Between Periodontal Diseases and Adverse Pregnancy Outcomes in Pregnant Women: A Systematic Review of Randomized Clinical Trials. Cureus.

[2] Beck J.D., Offenbacher S. (2005). Systemic effects of periodontitis: Epidemiology of periodontal disease and cardiovascular disease. J. Periodontol..

[3] Surya J.P. (2011). Causal relationship between periodontitis and chronic obstructive pulmonary disease. J. Indian Soc. Periodontol..

[4] AlSharief M., Alabdurubalnabi E. (2023). Periodontal Pathogens and Adverse Pregnancy Outcomes: A Narrative Review. Life.

[5] Chowdhury S.F., Islam M.N. (2021). Periodontal diseases among pregnant women attending an antenatal clinic at Dhaka, Bangladesh. J. Oral Res..

[6] Nannan M., Xiaoping L., Ying J. (2022). Periodontal disease in pregnancy and adverse pregnancy outcomes: Progress in related mechanisms and management strategies. Front. Med..

[7] Shanker R.K., Mohamed M., Hegle S., Kumar M.S. (2013). Influence of personality traits on gingival health. J. Indian Soc. Periodontal..

[8] Wu M., Chen S.W., Jiang S.Y. (2015). Relationship between gingival inflammation and pregnancy. Mediators Inflamm..

[9] Pozo E., Mesa F., Ikram M.H., Puertas A., Torrecillas-Martínez L., Ortega-Oller I., Magán-Fernández A., Rodríguez-Martínez M.D., Padial-Molina M., Sánchez-Fernández E. (2016). Preterm birth and/or low birth weight are associated with periodontal disease and the increased placental immunohistochemical expression of inflammatory markers. Histol. Histopathol..

[10] Ren H., Du M. (2017). Role of Maternal Periodontitis in Preterm Birth. Front. Immunol..

[11] Usin M.M., Menso J., Rodríguez V.I., Gonzalez A., Tabares S., Parodi R., Sembaj A. (2016). Association between maternal periodontitis and preterm and/or low birth weight infants in normal pregnancies. J. Matern. Fetal Neonatal. Med..

[12] Uwambaye P., Munyanshongore C., Rulisa S., Shiau H., Nuhu A., Kerr M.S. (2021). Assessing the association between periodontitis and premature birth: A case control study. BMC Pregnancy Childbirth.

[13] Minić I., Pejčić A. (2019). Parodontopathy as a Risk Factor for Premature Birth. Dentist.

[14] Papapanou P.N., Sanz M., Buduneli N., Dietrich T., Feres M., Fine D.H., Flemmig T.F., Garcia R., Giannobile W.V., Graziani F. (2018). Periodontitis: Consensus report of workgroup 2 of the 2017 World Workshop on the Classification of Periodontal and Peri-Implant Diseases and Conditions. J. Periodontol..

[15] Starzynska A., Wychowanski P., Nowak M., Sobocki B.K., Jereczek-Fossa B.A., Słupecka-Ziemilska M. (2022). Association between Maternal Periodontitis and Development of Systematic Diseases in Offspring. Int. J. Mol. Sci..

[16] Offenbacher S., Beck J.D., Lieff S., Slade G. (1998). Role of periodontitis in systemic health: Spontaneous preterm birth. J Dent. Educ..

[17] Williams R.C., Offenbacher S. (2000). Periodontal medicine: The emergence of a new branch of periodontology. Periodontol 2000.

[18] Tellapragada C., Eshwara V.K., Bhat P., Acharya S., Kamath A., Bhat S., Rao C., Nayak S., Mukhopadhyay C. (2016). Risk factors for preterm birth and low birth weight among pregnant Indian women: A hospital-based prospective study. J. Prev. Med. Public Health.

[19] Thakur R.K., Yadav B.K., Sultana R., Afridi S.K., Das D., Sahoo S.K. (2020). Influence of Periodontal Infection as a Possible Risk Factor for Preterm Low Birth Weight. J. Pharm. Bioallied. Sci..

[20] Ye C., Xia Z., Tang J., Khemwong T., Kapila Y., Kuraji R., Ping H., Wu Y., Kobayashi H. (2020). Unculturable and culturable periodontal-related bacteria are associated with periodontal inflammation during pregnancy and with preterm low birth weight delivery. Sci. Rep..

[21] WHO (2014). Global Nutrition Targets 2025: Low Birth Weight Policy Brief.

[22] Perin J., Mulick A., Yeung D., Villavicencio F., Lopez G., Strong K.L., Prieto-Merino D., Cousens S., Black P.R.E., Liu L. (2022). Global, regional, and national causes of under-5 mortality in 2000-19: An updated systematic analysis with implications for the Sustainable Development Goals. Lancet Child Adolesc. Health.

[23] Yankov Y.G., Bocheva Y. (2023). Comparative Characterization of Procalcitonin (Sensitivity, Specificity, Predictability, and Cut-Off Reference Values) as a Marker of Inflammation in Odontogenic Abscesses of the Head and Neck in the Female Population. Cureus.

[24] Acharya A.B., Shetty I.P., Jain S., Padakannaya I., Acharya S., Shettar L., Thakur S. (2019). Neutrophil-to-lymphocyte ratio and platelet-to-lymphocyte ratio in chronic periodontitis before and after nonsurgical therapy. J. Indian Soc. Periodontol..

[25] Desvarieux M., Demmer R.T., Jacobs D.R., Rundek T., Boden-Albala B., Sacco R.L., Papapanou P.N. (2010). Periodontal bacteria and hypertension: The oral infections and vascular disease epidemiology study (INVEST). J. Hypertens..

[26] Zhao L.Y., Yang D.D., Ma X.K., Liu M.M., Wu D.H., Zhang X.P., Ruan D.Y., Lin J.X., Wen J.Y., Chen J. (2019). The Prognostic Value of aspartate aminotransferase to lymphocyte ratio and systemic immune-inflammation index for Overall Survival of Hepatocellular Carcinoma Patients Treated with palliative Treatments. J. Cancer.

[27] Ishimine N., Honda T., Yoshizawa A., Kawasaki K., Sugano M., Kobayashi Y., Matsumoto T. (2013). Combination of White Blood Cell Count and Left Shift Level Real-Timely Reflects a Course of Bacterial Infection. J. Clin. Lab. Anal..

[28] Lee J.S., Kim N.Y., Na S.H., Youn Y.H., Shin C.S. (2018). Reference values of neutrophil-lymphocyte ratio, lymphocyte-monocyte ratio, platelet-lymphocyte ratio, and mean platelet volume in healthy adults in South Korea. Medicine.

[29] Hope C.K., Wang Q., Burnside G., Adeyemi A.A., Quenby S., Smith P.W., Higham S.M., Whitworth M. (2014). Assessing the Association between Oral Hygiene and Preterm Birth by Quantitative Light-Induced Fluorescence. Sci. World J..

[30] World Health Organization (WHO) (2013). Oral Health Survey. Basic Methods.

[31] Petersen P.E., Bourgeois D., Ogawa H., Estupinan-Day S., Ndiaye C. (2005). The global burden of oral diseases and risks to oral health. Bull. World Health Organ..

[32] Solari N., Salas L., Tabares S., Rosella C., Usin M.M., Sembaj A. (2023). Immunological and Bacteriological Monitoring of Periodontal Tissue in Pregnancy.-ODOVTOS-Int. J. Dental Sc..

[33] Amparo G.L., De Avila J., Castillo D.M., Montenegro D.A., Trujillo T.G., Suárez L.J., Lafaurie G.I. (2020). Porphyromonas gingivalis Placental Atopobiosis and Inflammatory Responses in Women with Adverse Pregnancy Outcomes. Front. Microbiol..

[34] Könönen E., Dareen F., Gursoy U.K., Gursoy M. (2022). *Prevotella* species as oral residents and infectious agents with potential impact on systemic conditions. J. Oral Microbiol..

[35] Suzuki N., Yoneda M., Hirofujj T. (2013). Mixed red-complex bacterial infection in periodontitis. Int. J. Dent..

[36] Vidmar Šimic M., Maver A., Zimani A.N., Hočevar K., Peterlin B., Kovanda A., Premru-Sršen T. (2023). Oral microbiome and preterm birth. Front. Med..

[37] Urushiyama D., Suda W., Ohnishi E., Araki R., Kiyoshima C., Kurakazu M., Sanui A., Yotsumoto F., Murata M., Nabeshima K. (2017). Microbiome profile of the amniotic fluid as a predictive biomarker of perinatal outcome. Sci. Rep..

